# Well under control: Control demand changes are sufficient for metacontrol

**DOI:** 10.3389/fpsyg.2022.1032304

**Published:** 2022-12-01

**Authors:** Moon Sun Kang, Chiu Yu-Chin

**Affiliations:** Department of Psychological Sciences, Purdue University, West Lafayette, IN, United States

**Keywords:** Metacontrol, cognitive control, control adaptation, emotion, affective processing

## Abstract

Metacontrol arises from the efficient retrieval of cognitive control by environmental cues that are predictive of the upcoming control demands. Previous studies have demonstrated that proactive and reactive metacontrol can be indexed by a list-wide switch probability (LWSP) and an item-specific switch probability (ISSP) effect, respectively. However, what triggers metacontrol in the first place has not been clearly articulated. While a “mere-experience” hypothesis attributes metacontrol to changes in control demands, an “affective-signaling” hypothesis suggests that high control demands are aversive and aversiveness drives metacontrol. In two experiments, we adjudicated between these hypotheses by considering the modes of metacontrol (proactive vs. reactive) and temporal dynamics of background valence (sustained vs. transient and positive vs. negative). We induced metacontrol (proactive or reactive) in a task-switching paradigm and created background valence by using positive and negative images as stimuli. With valence being an irrelevant aspect of the task, the design allows us to test whether (task-irrelevant) background valence would modulate metacontrol. While we were able to replicate the LWSP effect in Experiment 1 and the ISSP effect in Experiment 2, we did not find valence modulating either effect, regardless of the background valence being a sustained (Experiment 1) or a transient one (Experiment 2). These findings together suggest that negative valence (i.e., aversiveness) does not necessarily benefit metacontrol, and control demand variations are sufficient to induce metacontrol.

## Introduction

Cognitive control plays a critical role in coordinating sensory inputs, attention, and actions in line with our internal goals by focusing on possibly weak but goal-related properties while suppressing strong but goal-irrelevant ones (Miller and Cohen, 2001; Egner, 2017; Braem et al., 2019). One way that researchers have examined cognitive control is by using a task-switching paradigm where participants are required to switch between two categorization tasks that differ in their stimulus–response mappings (Jersild, 1927). A typical task-switching paradigm includes two different types of trial transition. A switch trial requires one to apply a different categorization task compared with the previous trial in a current trial, and a repeat trial requires one to apply the same categorization task in a current trial. By contrasting switch and repeat trials, the task-switching paradigm models cognitive control because of their difference in control demands. Such control-demand difference is reflected by longer response times and higher error rates on switch trials than on repeat trials, denoted as “switch costs.” Several non-mutually exclusive sources of switch costs have been posited, including task-set reconfiguration, transient task-set inertia, and associative retrieval accounts (Monsell, 2003; Waszak et al., 2003; Yeung and Monsell, 2003). Some researchers attribute switch costs to switch trials’ demands for retrieving a different task-set (e.g., a different stimulus–response mapping) from the previous trial, meaning that task-set reconfiguration on switch trials incurs switch costs (Monsell, 2003). Others attribute switch costs to demands for controlling task-set associations (Allport et al., 1994; Waszak et al., 2003), such as inhibiting long-term priming of previous associations that are now irrelevant or retrieving previously inhibited associations that are now relevant (see Monsell, 2003 for review). Likely, switch costs reflect some or all of these sources. The consensus is that switch costs nonetheless reflect a demand difference in task-set updating processes between switch and repeat trials. Most importantly, switch costs are meaningful and useful in examining how cognitive control relates to other cognitive and non-cognitive (e.g., affective) processes.

Traditionally, cognitive control has been considered strictly as a top-down process (Posner and Snyder, 1975; Cohen, 2017). However, recent studies suggested that control operations can be triggered by contextual cues bottom-up, indicating an automatic aspect of cognitive control (Bugg and Crump, 2012). One of the core benefits of cognitive control is its flexibility whereby new responses can be selected in accordance with an updated task-set or goal, as opposed to relying on rigid stimulus–response associations (e.g., habits). However, flexibility comes with a cost and entails expending effort (Kool et al., 2010; Westbrook and Braver, 2015). On the other hand, “automatic control” (Jacoby et al., 2003) represents an intriguing middle-ground whereby a particular control state (e.g., frequent/infrequent task-set updates) becomes associated with a context and is subsequently retrieved in that context (Braem and Egner, 2018; Chiu, 2019; Chiu and Egner, 2019). Automatic control is thought to be achieved by simply subjecting control to associative learning (Abrahamse et al., 2016). More specifically, as particular control co-occurs in a particular context repeatedly, the two become linked, forming “context-control” associations. Later on, the context acts as a *cue* that triggers the retrieval of the associated control state and the instantiation of a proper control operation. The bottom-up retrieval of the control state through the “context-control” associations has been referred to as a “metacontrol” process (Goschke, 2013; Hommel, 2015; Fröber and Dreisbach, 2020; Liu and Yeung, 2020). That is, when a certain contextual cue repeatedly predicts similar demands for task-set updating, metacontrol enables fast retrieval of the appropriate control state, which then facilitates efficient instantiation of the appropriate control operation. Metacontrol is a form of control adjustment (i.e., adaptive control) in that appropriate control states are instantiated dependent on different contexts.

### Two distinct modes of metacontrol

Based on a body of evidence demonstrating that metacontrol is mediated by different types of cues (e.g., location-, list-, item-based cues), researchers have proposed that there are at least two modes of metacontrol with distinct temporal characteristics (Gonthier et al., 2016; Braem et al., 2019): (1) proactive, sustained metacontrol that is maintained across a block (or “list”) of all trials/stimuli and can be instantiated prior to a trial’s onset; (2) reactive, transient metacontrol that can only be instantiated after a trial’s onset and can only be instantiated on specific trials/stimuli.

To demonstrate *proactive* metacontrol, studies have employed diagnostic items that are not switch-probability biased and mixed them with inducer items that are switch-probability biased. Although the diagnostic items were switch-unbiased themselves, the diagnostic items’ switch costs were reduced in a *list* with frequent switch trials as compared with those in a list with rare switch trials (Dreisbach and Haider, 2006; Monsell and Mizon, 2006; Schneider and Logan, 2006; Mayr et al., 2013; Kang and Chiu, 2021). The pattern is referred to as a “list-wide” switch probability (LWSP) effect. The “list-wide” description emphasizes that when a particular control state (e.g., high or low switch readiness) is consistently required by inducer items, it is maintained across all items in the same list, even if the item itself is not associated with frequent switching. That is, greater switch readiness (i.e., smaller switch costs) is maintained in the list with frequent switching, especially in switch-probability unbiased diagnostic items. The LWSP effect on switch-probability unbiased items (i.e., diagnostic items) highlights that the LWSP effect arises from proactive processing.

On the other hand, *reactive* metacontrol has been demonstrated by an “item-specific switch probability” (ISSP) effect where reduced switch costs are seen in stimuli (or *items*) associated with a high probability of switching compared with items associated with a low probability of switching (Leboe et al., 2008; Chiu and Egner, 2017). As the high and low switch probability items are presented together in a list with a 50% chance of switching, the “item-specific” description emphasizes that the appropriate control state (which varies between items) cannot be prepared prior to recognizing which item is which, and the appropriate control state has to be retrieved on a trial-by-trial (or item-by-item) basis after the onset of a trial. Therefore, the ISSP effect has been interpreted as reflecting transient retrieval of greater switch readiness on high switch probability items than on low switch probability ones.

Recently, we successfully replicated proactive and reactive metacontrol using a within-subjects design in a single experimental session (Kang and Chiu, 2021). Importantly, we demonstrated proactive metacontrol with the LWSP effect in diagnostic items (i.e., switch probability unbiased, 50% switch probability items) and reactive metacontrol with the ISSP effect in a switch probability unbiased list. The finding of the two distinct modes of metacontrol is well aligned with the prominent dual mechanisms of control (DMC) framework within which cognitive control is conceptualized as operating in proactive or reactive modes (Braver, 2012). While proactive control preempts potential conflicts, reactive control deals with conflicts in a late correction manner after recognizing the need to do so. Similarly, proactive and reactive metacontrol appear to inherit such temporal characteristics of cognitive control and simply integrate learning and memory retrieval to achieve optimal responses in a context-sensitive manner (Egner, 2014).

### Triggers of metacontrol

The LWSP and ISSP effects reflect “metacontrol,” or how our cognitive system adapts to various demands imposed by the environment *via* engaging proactive vs. reactive processing mechanisms, respectively. However, what exactly “triggers” these control adjustments (e.g., metacontrol) in the first place remains unresolved. A “mere-experience” hypothesis would suggest that the LWSP and the ISSP effects are triggered by the mere detection and experience of changes in control demand. This hypothesis is based on the classic conflict monitoring theory (Botvinick et al., 2001; Botvinick M. et al., 2004; Botvinick M. M. et al., 2004), suggesting that the mere detection and experience of conflicts will trigger control adjustment (i.e., metacontrol). The conflict monitoring theory is well-supported by the findings of the so-called congruency sequence effects (CSE). As an example, Gratton et al. (1992) employed a flanker task where a directional response is required according to a target (i.e., a centered arrow). The target was surrounded by non-target distractors which demanded either the same response (i.e., a congruent trial, →→→→→) or an opposite (i.e., an incongruent trial, ←←→←←) response. Due to distractors priming a different response than the target, incongruent trials have higher amounts of response conflict, typically resulting in longer response times and higher error rates. The performance difference between incongruent and congruent trials is referred to as the congruency effect. The size of the congruency effect, therefore, indexes the amount of conflict control employed. Beyond basic congruency effects, interestingly, Gratton et al. (1992) demonstrated that the congruency effect was reduced, following incongruent trials (e.g., →→←→→ on trial *n − 1*) than following congruent trials (e.g., →→→→→ on trial *n − 1*). The smaller congruency effect or the CSE is interpreted as a result of an increased conflict control on trial *n* due to experiencing conflicts on trial *n − 1*. We reason that, even though the LWSP and the ISSP effects reflect regulation of task-set control over a broader time span (i.e., more than two consecutive trials), the same underlying mechanism should apply to these situations. Therefore, according to the mere-experience hypothesis, we expect that the list-wide/item-specific switch probability manipulations should trigger metacontrol and produce the LWSP and the ISSP effects.

On the other hand, the “affective-signaling” hypothesis suggests that experiencing conflicts is intrinsically aversive and the induced aversiveness is the main driver of metacontrol (Dreisbach et al., 2018, 2019; Vermeylen et al., 2019; Dignath et al., 2020). The affective-signaling hypothesis was indeed supported by a couple of studies that directly tested whether conflicts *per se* are aversive. For example, Braem et al. (2017) examined the activation in the anterior cingulate cortex (ACC) when participants were viewing pictures with negative or positive valence. They adopted a task-switching paradigm where participants switched between two tasks. The congruent trials were non-conflict trials because the stimuli required the same key press in both tasks, while the incongruent trials were conflict trials because the stimuli on those trials required different key presses in the two tasks. They found reduced ACC activation for negative pictures following incongruent trials than those following congruent trials. Based on the repetition suppression idea (i.e., the activation of the same brain areas is reduced when they are repeatedly activated), this key finding was taken as evidence that incongruent trials (i.e., conflict trials) are aversive and are capable of activating the ACC—which attenuates the ACC’s subsequent activation to the following negative pictures. Also supporting the affective-signaling hypothesis, Fröber et al. (2017) found that only in conflict trials which participants evaluated as aversive, was there physiological evidence of metacontrol in the form of suppression of automatic response activation. Given that switch trials are also high-conflict trials (Vermeylen et al., 2019), we reason that the affective-signaling hypothesis is applicable here: The LWSP/ISSP effects might be driven by the aversiveness in the high switch probability condition (in contrast with the low one), because it contains a greater amount of conflicts. Note that the “affective-signaling” hypothesis we refer to focuses on conflict-triggered aversiveness which is well aligned with the previous studies (e.g., Dreisbach et al., 2018, 2019; Vermeylen et al., 2019) rather than any type of aversiveness.

In fact, many prior studies have asked very similar questions as ours. But, the results are rather mixed in the literature regarding on how background valence modulates metacontrol. Some studies did not find metacontrol to be modulated by affective backgrounds (e.g., Cañadas et al., 2016; Zhang et al., 2019). However, some studies have found negative background enhancing metacontrol, possibly due to hyper-aversiveness when conflicts are processed in a negative valence background (van Steenbergen et al., 2009, 2010). Whereas, others have found positive valence background enhancing metacontrol, presumably due to loomed aversiveness as a result of having the opposite valence between the background and the conflicts *per se* (Dreisbach and Haider, 2006; Dreisbach et al., 2018, 2019). Admittedly, van Steenbergen et al. (2009, 2010) and Dreisbach et al. (2019) adopted very different cognitive paradigms, which could explain the inconsistencies. Nonetheless, we notice two factors that might have been overlooked in these prior studies but are worth taking into account.

First, it is important to consider that there are two distinct modes of metacontrol. As mentioned earlier in the introduction, while both the LWSP and ISSP effects reflect metacontrol, they likely reflect different mechanisms in action. Therefore, valence manipulation might interact with these mechanisms differently. The LWSP effect likely relies more on proactive processing, where a predetermined control state is applied to all items in the same list, giving less attention to the items themselves. Whereas, the ISSP effect relies predominantly on reactive processing because the items need to be recognized before the appropriate item-associated control states could be retrieved. In addition, neural substrates responsible for proactive and reactive metacontrol are thought to be dissociable based on the evidence that the dorsolateral prefrontal cortex supports the proactive mode of cognitive control (Constantinidis et al., 2001; Wallis et al., 2001) and the anterior cingulate cortex (ACC) supports the reactive mode (Cohen et al., 2000; MacDonald et al., 2000; Botvinick et al., 2001). Since the ACC is often activated while processing negative affect and pain (Rainville et al., 1997; Shackman et al., 2011)—given the shared processing structure—negative affect might enhance reactive metacontrol more easily as compared to proactive metacontrol. Note that studies without modulation effects of metacontrol by affective backgrounds (e.g., Cañadas et al., 2016; Zhang et al., 2019) induced proactive processing by presenting specific affective backgrounds associated with greater control recruitments before stimulus onsets. By considering two modes of metacontrol, we can examine if affective backgrounds interact with proactive and reactive metacontrol in different manners. Second, the temporal dynamics of the background valence (in addition to the values of valence, i.e., positive vs. negative) should also be considered. The background valence is much more “sustained” when inducing a positive vs. negative mood in each participant, as in the van Steenbergen et al. (2010)’s study. By contrast, when valence is manipulated on a trial-by-trial basis (e.g., van Steenbergen et al., 2009) or on an item-by-item basis (e.g., Dreisbach et al., 2019), the background valence is more transient in nature. By examining the effect of sustained, transient affective background on metacontrol, we can answer if a temporarily distinct affective background would result in different effects in terms of modulating metacontrol.

### The current experiments

We considered two modes of metacontrol and took into consideration that proactive and reactive metacontrol possibly interact differently with affective backgrounds. In addition, we considered that previous studies adopted temporally different affective backgrounds such that affective background was present in a sustained manner or on a trial-by-trial basis. With these two considerations in mind, we designed the current study to systematically examine whether background valence interacts with metacontrol in two experiments using a task switching paradigm. In particular, we based off a cued-task switching paradigm where we have successfully observed the LWSP and ISSP effect when using neutral images (Kang and Chiu, 2021). Instead of using neutral images, we employed positively and negatively valenced images to see if metacontrol is modulated by the background valence (positive or negative) in which conflicts are processed. The design had benefits in that it ensures to induce two metacontrol modes. Furthermore, switch trials are aversive, like the incongruent trials in conflict control paradigms (Vermeylen et al., 2019). In both experiments, we used positively-and negatively-rated (both by the database contributors and by our Experiment 2 participants) images as task stimuli to create positive and negative backgrounds. In Experiment 1, we examined the *sustained* background valence effect on metacontrol. We induced both proactive and reactive metacontrol in each subject but manipulated valence *between-subjects*. Namely, a positive-valence group was presented exclusively with the positive stimuli and a negative-valence group was presented exclusively with the negative stimuli. The positive-valence group was exposed to the positive valence in a sustained manner, while the negative-valence group was exposed to the negative one. In Experiment 2, we examined the *transient* background valence effect on metacontrol. We induced only reactive metacontrol and manipulated valence *between-items (or stimuli).* By noting that reactive metacontrol relies on item types (i.e., high vs. low switch probability items), we paired item types and valence, while holding the overall background valence neutral within each participant. Specifically, one group of subjects performed the task with high switch probability items that are positively valenced, and low switch probability items that are negatively valenced. The other group of subjects received the opposite pairings.

In summary, in Experiment 1, we tested if the LWSP/ISSP effects are modulated by *sustained* background valence whereas, in Experiment 2, we tested if the ISSP effect is modulated by *transient* background valence. According to the mere-experience hypothesis, metacontrol should be sufficiently triggered by changes in control demands. We, therefore, expect that valence should not interact with the LWSP/ISSP effects. However, if metacontrol is triggered by aversiveness, as suggested by the affective-signaling hypothesis, background valence should interact with both the LWSP/ISSP effects. To support the affective-signaling hypothesis, we expect to observe the larger LWSP/ISSP effects in the negative (vs. positive) background due to the enhanced aversiveness in the negative background. It is based on the rationale that the negative affect would incur greater cognitive modulation on high vs. low conflict lists/items due to enhanced control on high conflict lists/items experienced in the negative background. To preview our results, we did not find the sustained background valence interacting with the LWSP and the ISSP effect. Nor did we find the transient background valence interacting with the ISSP effect. Our results support the mere-experience hypothesis that the control demand variations are sufficient to trigger metacontrol. However, the exploratory analysis revealed some support for the affective-signaling hypothesis in that we observed enhanced reactive metacontrol in the sustained, negative background valence (than in the sustained, positive one). But this was only the case among female participants. Implications for exploring gender differences in the domain of affect-metacontrol interaction will be further discussed in General discussion.

## Experiment 1

### Method

#### Participants

Two hundred and eighty-three undergraduate students (*M_age_* = 18.9, *SD_age_* = 1.31, 142 females, 141 males) provided informed consent to participate in this study in return for 2-course credits. The participant’s gender was determined by a forced-choice binary question (male or female). The study was approved by the Purdue University Institutional Review Board. The minimum sample size to detect the ISSP effect was estimated to be 140 by a simulation procedure with the following parameter: type I error = 0.05, power = 0.90, switch costs: M_low switch items_ = 61.38, SD_low switch items_ = 41.41, M_high switch items_ = 72.59, SD_high switch items_ = 44.69 (Experiment 1, Kang and Chiu, 2021). The simulation procedure was implemented in R by randomly drawing samples from a multivariate normal distribution built by the switch costs on low switch items and switch costs on high switch items obtained in our previous study. We checked the smallest sample size which produces an effect size of 0.90. We doubled this number because we have two valence conditions (positive vs. negative), resulting in a sample size of 280. Data from 272 (138 females, 134 males) participants were analyzed and reported below after excluding 11 participants due to their overall mean accuracy being outside of the group mean ± 2 SD. The 1st and 3rd quartile accuracy scores from the sample size 272 were 76% and 89%. Experiment 1 was not pre-registered and was conducted first. Although we did not pre-register for this study, the sample size was planned in a way to have sufficient power to detect the ISSP effect.

#### Stimuli

We used full-color positive- (*M*_valence_ = 7.2, *M*_arousal_ = 4.6) and negative- (*M*_valence_ = 3.5, *M*_arousal_ = 6.0) valence images, which were developed and rated by the Nencki Affective Picture System (NAPS) contributors (Marchewka et al., 2014). The rating results are accessible upon request.^1^ We selected a subset of images from the NAPS database (i.e., a face-image set) that are applicable to our two categorization tasks (i.e., gender/age tasks) with proper compositions such that one person is centered in the middle of each image. All of the images contain a person and our metacontrol cued-task switching paradigm required participants to switch between categorizing the subject according to his/her age (i.e., a child vs. an adult) or according to his/her gender (i.e., a female vs. a male). In line with the two categorization tasks, the images belonged to one of the four stimulus categories, a female child, a female adult, a male child, and a male adult. For each participant, eight images (4 from two of the stimulus categories) were used in the main experiment. The images from each category were randomly chosen for each participant. A different set of eight images (2 from each of the four stimulus categories) was used exclusively for the practice. All the images in the practice and the main experiment trials were displayed with a size of 499 pixels in width and 500 pixels in height.

#### Design and Procedure

The main experiment included three lists of 240 trials with a low, medium, and high *list-wide switch probability*. The high and the low switch probability lists (with the order counterbalanced across participants) were administered first. The high and the low switch probability lists were designed to induce proactive metacontrol and the medium (50%) switch probability list was designed to induce reactive metacontrol.

The high/low switch probability list included a different number of switch and repeat trials according to its list-wide switch probability. In the low switch probability list, half of the items were associated with a 10% chance of switching (2 items; 108 repeat trials; 12 switch trials), and the other half were associated with a 50% chance of switching (2 items; 60 switch trials; 60 repeat trials), resulting in a 30% chance of switching at the list level. In the high switch probability list, half of the items were associated with a 90% chance of switching (2 items; 12 repeat trials; 108 switch trials), and the other half were associated with a 50% chance of switching (2 items; 60 repeat trials; 60 switch trials), resulting in a 70% chance of switching at the list level. The items associated with a 50% chance of switching were referred to as *diagnostic* items (i.e., switch probability-unbiased items), while the items associated with a 10% or 90% chance of switching were referred to as *inducer* items (i.e., switch probability-biased items). The diagnostic and the inducer items in the low and high switch probability lists were all unique and did not overlap between lists (i.e., a total of 8 unique items across the two lists). *Proactive metacontrol* was assessed with the *diagnostic* items presented in the low vs. high list-wide switch probability lists.

The medium switch probability list included an equal number of switch and repeat trials but has an item-specific switch probability (ISSP) design. Namely, half of the items were associated with a 10% chance of switching (2 inducer items in the low LWSP list; 108 repeat trials; 12 switch trials), and the other half were associated with a 90% chance of switching (2 inducer items in the high LWSP list; 12 repeat trials; 108 switch trials). These items were the same inducer items from the low and the high switch probability lists. We reused these inducer items to facilitate item-specific learning and because of this, the medium switch probability list was administered last. *Reactive metacontrol* was assessed with the 10 and 90% switch probability items presented in the unbiased, medium (50%) list-wide switch probability list. The trials in each list were randomized with the constraint that each item appeared in the two tasks roughly equally (i.e., no more than 2 trials in difference). This procedure ensured that each item was roughly associated with the two tasks/responses equally, avoiding the possibility of strong stimulus–response learning confounding the results (Schmidt and Besner, 2008).

**Figure 1:**
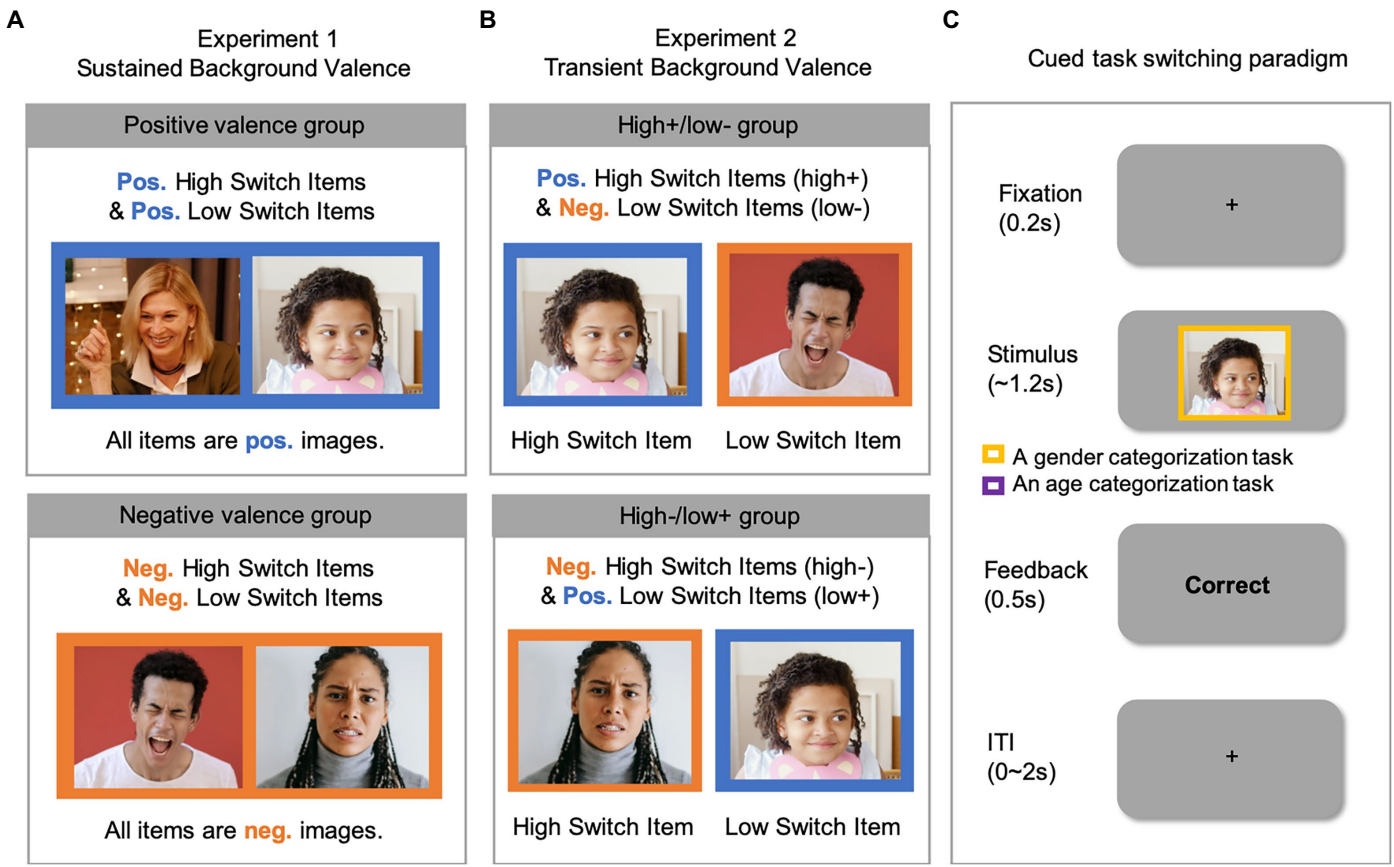
The overall background valence manipulation for **(A)** Experiment 1 and **(B)** Experiment 2 and **(C)** an example trial in the cued task-switching paradigm. Note that the images (selected from https://www.pexels.com/) shown in this figure are for illustration purposes only. Please see Method for detailed descriptions regarding the source and the stimulus selection procedure of the images used in the experiment. Neg., Negative valence; Pos., Positive valence.

Most importantly, our key variable of *sustained background valence* was manipulated *between-subjects*. Specifically, the positive-valence group saw positively-valenced images as stimuli in all three lists (Figure 1A, top row). The negative-valence group saw negatively-valenced images as stimuli in all three lists (Figure 1A, bottom row). Participants were instructed to categorize the person in each image by gender or by age cued by the color of a frame (blue or red) surrounding the stimulus on each trial. The color-to-task mapping was counterbalanced across participants.

For each participant, 8 images (4 from two of the stimulus categories) were used in the main experiment. The two stimulus categories were both “response incompatible” according to each participant’s stimulus–response assignment (i.e., producing different responses in the two categorization tasks). Based on the logic that response compatible items are susceptible to lower-level stimulus–response learning (Schmidt and Besner, 2008), we avoided using response compatible items (i.e., producing the same response in the two categorization tasks). This design choice allowed us to maximize learning on each item (i.e., more trials per item) in Experiment 1 and fit both list-wide and item-specific manipulations in an 1.5-h experiment in order to examine the effect of valence on both proactive *and* reactive metacontrol. We have recently shown that this design produces similar results as one including both response compatible and incompatible items (Kang and Chiu, 2021; Experiment 2; see also Spinelli et al., 2019; Spinelli and Lupker, 2021; Spinelli and Lupker, 2022).

The image stimuli were presented for 1,200 ms after a 200 ms fixation. The image disappeared after responses if they were made within 1,200 ms and the rest time was filled with a blank screen. The stimulus presentation was followed by a 500 ms feedback and an inter-trial interval presented for a time that was randomly sampled from a range between 0 and 2,000 ms. The feedback was the word “correct” for correct responses, “incorrect” for incorrect responses, or “too late” for the absence of responses (Figure 1C). Two keys on a standard QWERTY keyboard, “V” and “N,” were designated as response keys. The stimulus category-to-response mapping was counterbalanced across participants. Before the main experiment, a practice session with unbiased switch probability was administered to familiarize participants with their designated stimulus–response mapping. In the practice trials, participants repeated the practice trials until they obtain a mean accuracy of 80% or higher. All the experiments were conducted online.

#### Data analysis

Before the main analysis that tested our hypothesis, we first checked if our paradigm was able to successfully induce proactive and reactive metacontrol. To this end, we calculated two scores to index the two different modes of metacontrol in each participant. To index proactive metacontrol, we first calculated switch costs (SC: switch - repeat) of each list (i.e., high, low switch probability list) and then the difference between the lists’ SC (SC_low list_ − SC_high list_). Note that we only used “diagnostic items” in this calculation, as shown before (e.g., Kang and Chiu, 2021), because proactive metacontrol should be manifested in all items, even in switch-probability ‘unbiased’ diagnostic items. Likewise, to index the reactive metacontrol, we first calculated each item’s (i.e., high, low switch probability items in the unbiased, medium switch probability list) switch costs and then the difference between the items’ SC. With the entire sample, we performed a one-sample *t*-test (against μ  = 0) on each score, which is equivalent to testing whether the LWSP/ISSP effect was significant.

Next, to address the main question of whether background valence modulates metacontrol, we then compared the metacontrol scores between the positive-valence group and the negative-valence group using a two-tailed two independent samples Welch’s unequal variances *t*-test. This comparison was done separately for the LWSP and the ISSP effect. We used Welch *t*-test statistics because they do not require homoscedasticity between groups.

All of the above analyses were performed on the response time (RT) and accuracy (ACC) separately. When calculating mean RT in each condition, we excluded trials with incorrect responses and with RT values beyond ±3 SD of each participant’s mean (all correct trials). Along with inferential statistics, we report descriptive statistics, e.g., means and standard deviations (SD), and Cohen’s *d* as measures of effect sizes.

In addition to the frequentist statistics, we also calculated and reported Bayes Factors (BF) using the “ttestBF” function in the “BayesFactor” package with a default prior scale (i.e., rcale = “medium”) in R. We reported in a form of BF_01,_ which is a ratio of evidence for the null hypothesis (H_0_) given data over evidence for the alternative hypothesis (H_1_) given data. A BF_01_ between 0 and 1 means negative evidence, between 1 and 3 means negligible evidence, and between 3 and 20 means substantial evidence for the null hypothesis (Kass and Raftery, 1995).

### Results

#### Proactive metacontrol

##### Response times

**Figure 2:**
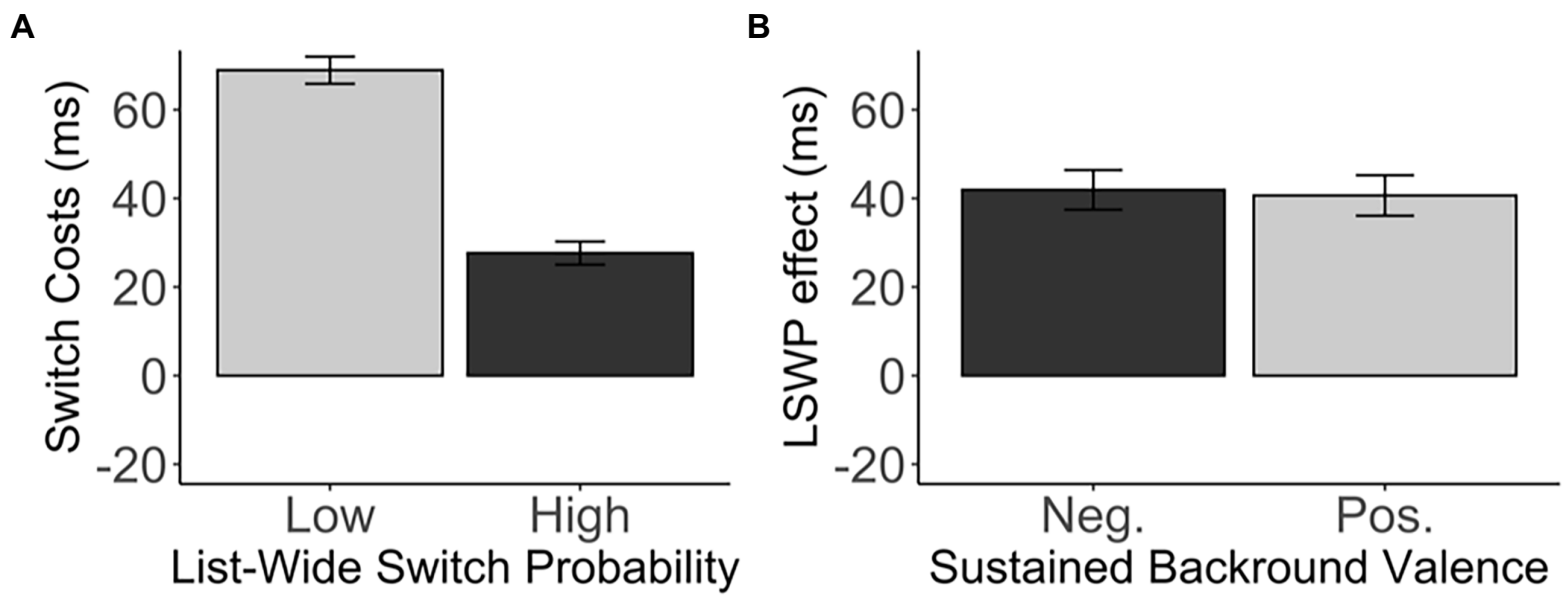
**(A)** The switch costs (in ms) of the diagnostic items in the low and high list-wide switch probability lists. **(B)** The LWSP effect (in ms) as a function of the sustained background valence. Error bars indicate the standard error of the mean. Neg., Negative valence; Pos., Positive valence.

As expected, our design successfully induced proactive metacontrol, replicating the LWSP effect (M = 41.27, SD = 52.72), *t*(271) = 12.91, *p* < 0.001, *d* = 0.78, 95% CI [34.98, 47.57], BF_01_ < 0.001 (Figure 2A). This effect was due to the smaller switch costs in the diagnostic items that were embedded in the high list-wide switch probability list (M = 27.66, SD = 43.07) than those embedded in the low list-wide switch probability list (M = 68.93, SD = 50.54). However, *sustained background valence* did not enhance nor decrease proactive metacontrol. There was no difference in the LWSP effect between the negative-valence group and the positive-valence group, *t*(269.98) = −0.20, *p* = 0.843, *d* = 0.02, 95% CI [−13.86, 11.33], BF_01_ = 7.37 (negative: M = 41.92, SD = 51.41; positive: M = 40.66, SD = 54.10). See Table 1 and Figure 2B.

**Table 1:** Mean response times (ms) with standard deviations in Experiment 1 as a function of background valence, switch probability, and transition.

Switch probability	Transition	Negative	Positive
		*N* = 132	*N* = 140
Low list	Repeat	682 (68)	675 (96)
	Switch	749 (84)	747 (108)
High list	Repeat	719 (80)	704 (89)
	Switch	743 (94)	735 (103)
Low items	Repeat	694 (83)	689 (104)
	Switch	723 (104)	726 (121)
High items	Repeat	704 (98)	686 (107)
	Switch	723 (95)	717 (114)

Low and high lists refer to the diagnostic items in the low and high list-wide switch probability lists. Low and high items refer to the low and high switch probability items in the medium (50%) list-wide switch probability list. N represents the sample size in each group.

##### Accuracy

Similar to RT, we replicated the LWSP effect (M = 6.77, SD = 12.11), *t*(271) = 9.22, *p* < 0.001, *d* = 0.56, 95% CI [5.33, 8.22], BF_01_ < 0.001. The switch costs were reduced in the diagnostic items embedded in the high list-wide switch probability list (M = 6.58, SD = 8.11) as compared to those embedded in the low list-wide switch probability list (M = 13.35, SD = 10.27). However, the LWSP effect was not modulated by *sustained background valence*, *t*(267.2) = 1.65, *p* = 0.10, *d* = 0.20, 95% CI [−0.47, 5.31], BF_01_ = 2.05 (negative: M = 5.53, SD = 12.33; positive: M = 7.95, SD = 11.81). See Table 2.

**Table 2:** Mean accuracy (%) with standard deviations in Experiment 1 as a function of background valence, switch probability, and transition.

Switch probability	Transition	Negative	Positive
		*N* = 132	*N* = 140
Low list	Repeat	86 (11)	86 (10)
	Switch	74 (14)	71 (14)
High list	Repeat	82 (12)	83 (10)
	Switch	75 (15)	76 (13)
Low items	Repeat	87 (12)	86 (11)
	Switch	83 (16)	80 (16)
High items	Repeat	88 (15)	88 (13)
	Switch	83 (14)	82 (13)

Low and high lists refer to the diagnostic items in the low and high list-wide switch probability lists. Low and high items refer to the low and high switch probability items in the medium (50%) list-wide switch probability list. N represents the sample size in each group.

#### Reactive metacontrol

##### Response times

**Figure 3:**
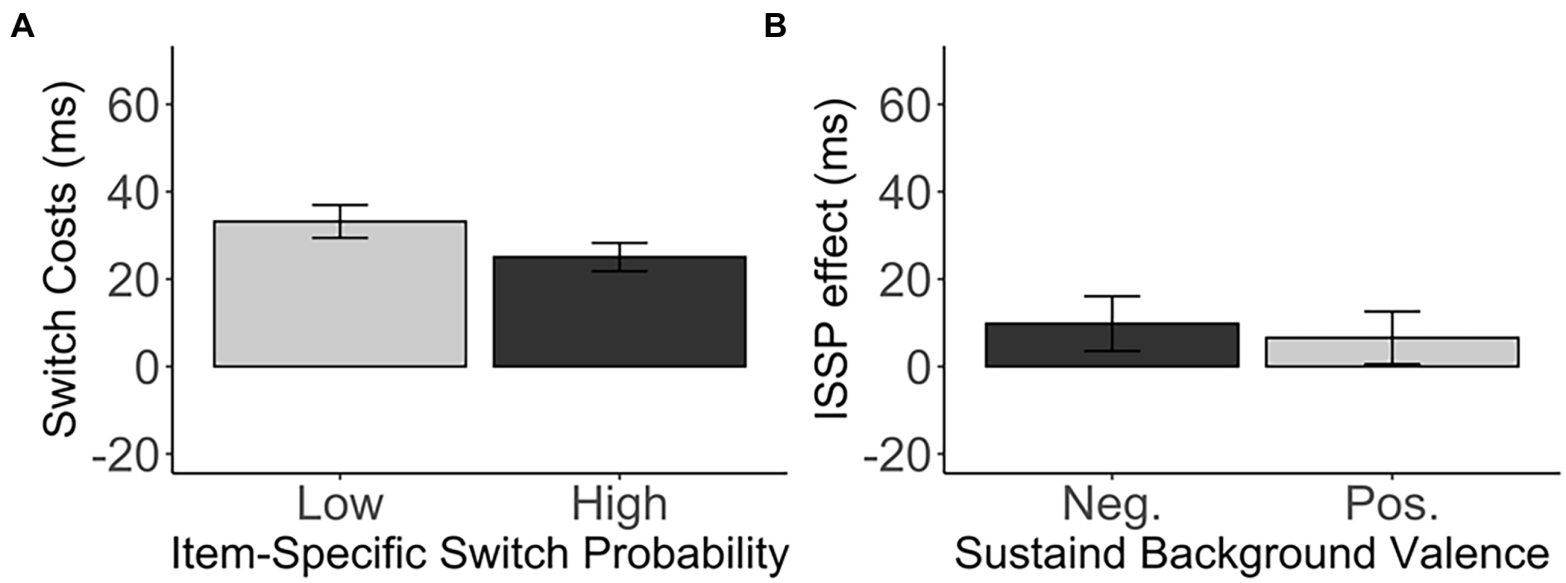
**(A)** The switch costs (in ms) of the low and high switch probability items in the medium, unbiased list-wide switch probability list. **(B)** The ISSP effect (in ms) as a function of the sustained background valence. Error bars indicate the standard error of the mean. Neg., Negative valence; Pos., Positive valence.

Unexpectedly, the ISSP effect (M = 8.15, SD = 71.47) did not reach significance, *t*(271) = 1.88, *p* = 0.061, *d* = 0.11, 95% CI [−0.38, 16.68], BF_01_ = 2.59 (Figure 3A), although numerically in the right direction. That is, switch costs in the high switch probability items (M = 25.05, SD = 53.35) were smaller than switch costs in the low switch probability items (M = 33.20, SD = 62.13) in the unbiased switch probability list.

Furthermore, we did not observe any difference in the ISSP effect between the negative and positive *sustained background valence*, *t*(268.68) = − 0.37, *p* = 0.709, *d* = 0.05, 95% CI [−20.35, 13.86], BF_01_ = 7.02 (negative: M = 9.82, SD = 71.99; positive: M = 6.57, SD = 71.20). See Table 1 and Figure 3B. The sustained background valence did not modulate the ISSP effect.

##### Accuracy

The ISSP effect was not significant, *t*(271) < 0.01, *p* = 0.994, *d* < 0.01, 95% CI [−1.59, 1.60], BF_01_ = 14.72, and neither modulated by *sustained background valence.* There was no difference in the ISSP effect between the negative-valence group and the positive-valence group, *t*(269.42) = 0.67, *p* = 0.501, *d* = 0.08, 95% CI [−2.09, 4.26], BF_01_ = 6.06 (negative: M = −0.55, SD = 12.61; positive: M = 0.53, SD = 14.01). See Table 2.

#### Exploratory analysis results

Since we failed to observe the ISSP effect which has been replicated elsewhere (Chiu and Egner, 2017; Kang and Chiu, 2021), we explored if an individual difference factor, i.e., sensitivity to negative affect, played a role here. Some studies notably demonstrated that female participants are more responsive to negative images (Wrase et al., 2003; Kemp et al., 2004; Codispoti et al., 2008). For instance, George et al. (1996) reported that female participants displayed greater activation (compared to male participants) in the bilateral anterior cingulate and the left medial prefrontal cortex when they are in a negative mood. Wrase et al. (2003) used the standardized valence images (i.e., International Affective Picture System, Center for the Study of Emotion and Attention, 1999) and found that the negative images induced stronger anterior and medial cingulate activation in female participants than in male participants. Thus, given that the ACC has been demonstrated to be responsible for affective processes including emotion regulation (Rainville et al., 1997; Shackman et al., 2011), one might suspect that females are more responsive to negative images than males. In addition to the greater neurological responses, self-reported arousal ratings were higher in female participants than in male ones (Center for the Study of Emotion and Attention, 1999). Since we did not have a way to index an individual’s sensitivity to negative valence, we employed participants’ self-reported gender as a categorical variable in our exploratory analysis. Doing so allowed us to compare with prior studies that included mostly female participants when examining interactions between valence and metacontrol (e.g., van Steenbergen et al., 2009, 2010; Dreisbach et al., 2018, 2019), perhaps due to convenience sampling.

Therefore, to investigate a possible gender difference in responsiveness to the negative images, we compared the item interaction scores between the positive-valence group and the negative-valence group, separately for the female and the male sample. Same as in the main analyses, we used a two-tailed two-independent samples Welch’s unequal variances *t*-test. To control for study-level type I error rate, we applied the Bonferroni corrections due to conducting two independent tests (i.e., one *t*-test each in female and male samples) for each list/item interaction score. Note that this exploratory analysis was initiated to explore an underlying mechanism for the ISSP effect failing to reach significance. However, with the LWSP effect, we also conducted the same exploratory analysis as outlined above. This additional analysis with the LWSP effect allowed us to investigate if gender also influenced how background valence might interact with proactive metacontrol.

Lastly, even though we did not plan to include gender as a between-subjects variable in our design, our data nonetheless could be analyzed by an omnibus 2 (item-specific/list-wide switch probability: low, high) × 2 (transition: repeat, switch) × 2 (background valence: positive, negative) × 2 (gender: female, male) mixed-design rmANOVA. Note that our sample size might be small for detecting higher-order interactions in this 4-way mixed effect ANOVA. Nonetheless, for completeness’ sake, we reported those results in the supplementary document at http://doi.org/10.17605/OSF.IO/2BDVC.

#### Proactive metacontrol

##### Response times

**Figure 4:**
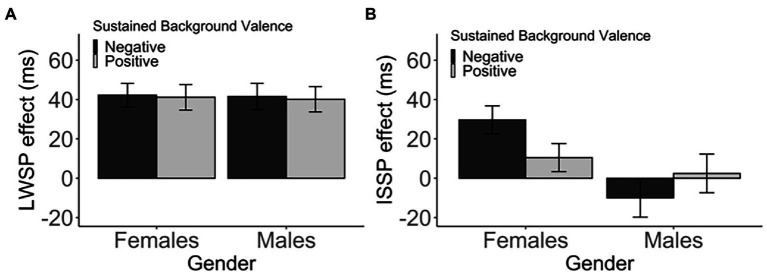
The **(A)** LWSP and **(B)** ISSP indicate the standard error of the mean.

In female participants, there was no difference in the LWSP effect between the two *sustained background valence*, *t*(135.77) = 0.12, *p* > 1 (corrected), *d* = 0.02, 95% CI [−16.44, 18.64], BF_01_ = 5.44 (negative: M = 42.26, SD = 48.74; positive: M = 41.16, SD = 55.45). Likewise, in male participants, there was no reliable difference, *t*(131.62) = 0.16, *p* > 1 (corrected), *d* = 0.03, 95% CI [−16.90, 19.82], BF_01_ = 5.35 (negative: M = 41.59, SD = 54.33; positive: M = 40.13, SD = 53.05). See Table 3 and Figure 4A. In both genders, the LWSP effect was neither enhanced nor decreased in the negative background as compared to the positive background.

**Table 3:** Mean response times (ms) with standard deviations in Experiment 1 as a function of background valence, switch probability, and transition separately in males and females.

		Negative		Positive	
Switch probability	Transition	Females	Males	Females	Males
		*N* = 66	*N* = 66	*N* = 72	*N* = 68
Low list	Repeat	692 (72)	673 (62)	675 (66)	676 (121)
	Switch	757 (84)	741 (83)	753 (69)	740 (138)
High list	Repeat	730 (76)	707 (82)	705 (69)	703 (106)
	Switch	752 (85)	734 (101)	742 (71)	727 (128)
Low items	Repeat	704 (70)	684 (94)	696 (59)	681 (137)
	Switch	748 (81)	698 (118)	738 (85)	713 (150)
High items	Repeat	721 (76)	686 (114)	697 (71)	675 (135)
	Switch	735 (75)	710 (111)	729 (66)	704 (148)

Low and high lists refer to the diagnostic items in the low and high list-wide switch probability lists. Low and high items refer to the low and high switch probability items in the medium (50%) list-wide switch probability list. *N* represents the sample size in each group.

##### Accuracy

The LWSP effect was neither modulated by *sustained background valence* in female participants, *t*(135.65) = −1.03, *p* = 0.612 (corrected), *d* = 0.18, 95% CI [−6.42, 2.03], BF_01_ = 3.40 (negative: M = 6.01, SD = 11.67; positive: M = 8.21, SD = 13.41), nor in male participants, *t*(121.48) = −1.31, *p* = 0.386 (corrected), *d* = 0.23, 95% CI [−6.60, 1.34], BF_01_ = 2.46 (negative: M = 5.04, SD = 13.04; positive: M = 7.67, SD = 9.93). See Table 4.

**Table 4:** Mean accuracy (%) with standard deviations in Experiment 1 as a function of background valence, switch probability, and transition separately in males and females.

		Negative		Positive	
Switch probability	Transition	Females	Males	Females	Males
		*N* = 66	*N* = 66	*N* = 72	*N* = 68
Low list	Repeat	85 (11)	86 (11)	86 (8)	85 (11)
	Switch	73 (14)	75 (14)	70 (13)	72 (14)
High list	Repeat	81 (12)	83 (12)	83 (10)	82 (11)
	Switch	74 (15)	76 (14)	76 (13)	76 (14)
Low items	Repeat	86 (13)	88 (12)	88 (8)	85 (13)
	Switch	82 (17)	85 (16)	80 (16)	79 (17)
High items	Repeat	87 (13)	88 (17)	88 (12)	88 (15)
	Switch	82 (13)	85 (14)	83 (11)	81 (14)

Low and high lists refer to the diagnostic items in the low and high list-wide switch probability lists. Low and high items refer to the low and high switch probability items in the medium (50%) list-wide switch probability list. *N* represents the sample size in each group.

#### Reactive metacontrol

##### Response times

Interestingly, although not statistically significant, there was a numerical tendency of the ISSP effect being modulated by *sustained background valence* in female participants, *t*(135.79) = 1.90, *p* = 0.118 (corrected), *d* = 0.32, 95% CI [−0.76, 39.11], BF_01_ = 1.07. The ISSP effect was numerically larger in the negative-valence group (M = 29.66, SD = 57.76) as compared to the positive-valence group (M = 10.48, SD = 60.64), More specifically, post-hoc *t*-tests revealed that the ISSP effect was significant in the negative-valence group, *t*(65) = 4.17, *p* < 0.001 (corrected), *d* = 0.51, BF_01_ < 0.01, but not in the positive-valence group, *t*(71) = 1.47, *p* = 0.294 (corrected), *d* = 0.17, BF_01_ = 2.78 (Figure 4B; Table 3). However, the ISSP effect was not modulated by sustained valence in male participants, *t*(131.99) = −0.90, *p* = 0.742 (corrected), *d* = 0.16, 95% CI [−39.89, 15.00], BF_01_ = 3.75 (negative: M = −10.02, SD = 79.44; positive: M = 2.43, SD = 81.17).

##### Accuracy

In female participants, there was no difference in the ISSP effect between the negative-valence group and the positive-valence group, *t*(135.61) = −1.41, *p* = 0.322 (corrected), *d* = 0.24, 95% CI [−7.92, 1.33], BF_01_ = 2.22 (negative: M = −0.84, SD = 13.49; positive: M = 2.45, SD = 13.96). Likewise, in male participants, there was no difference between the two valence groups, *t*(129.65) = 0.56, *p* > 1 (corrected), *d* = 0.10, 95% CI [−3.15, 5.63], BF_01_ = 4.70 (negative: M = −0.26, SD = 11.75; positive: M = −1.50, SD = 13.87). See Table 4.

### Discussion

In Experiment 1, we examined whether the LWSP/ISSP effects are modulated by sustained background valence. To this end, we used highly positively-and negatively-valenced stimuli in a metacontrol task-switching paradigm we established previously (with neutral stimuli; Kang and Chiu, 2021). We then compared the LWSP/ISSP effects between groups of participants that performed the categorization tasks with either positive or negative stimuli. Note that the valence background in Experiment 1’s design was a *sustained* one, as opposed to a *transient* one, because each participant was exposed to only one of the background valences throughout the experiment. We found that while the LWSP effect was replicated, it was not modulated by background valence. In other words, proactive metacontrol does not appear to be enhanced nor decreased in the sustained negative background where aversiveness might be experienced. Unlike the LWSP effect, we did not find a significant ISSP effect indexing reactive metacontrol. The ISSP effect was neither modulated by the sustained background valence such that the sustained negative background did not enhance or decrease the ISSP effect.

Surprised by not replicating the ISSP effect, we explored a possible gender difference in responsiveness to our valence manipulation. The post-hoc *t-*tests revealed that the ISSP effect was only significant in female participants that performed the categorization tasks with negatively-valenced stimuli. However, we did not observe such gender differences in the LWSP effect. Of worth noting is that the females’ ISSP effect from the sustained negative background group has an effect size of 0.51 (in Cohen’s d) which is numerically larger than any of our previous findings (Kang and Chiu, 2021: ~0.26; Chiu and Egner, 2017: ~0.28). We caution against making strong conclusions about this descriptive and observational finding. Nonetheless, the insignificant ISSP effect might have been driven by intriguing interactions worth following up in future studies. This result hinted that reactive metacontrol, in particular, may be enhanced by increased aversiveness. We will return to this finding in the general discussion section. Overall, the results in Experiment 1 together suggest that control demand variations are sufficient to induce proactive and reactive metacontrol.

## Experiment 2

Since reactive metacontrol relies on identifying an item-type (high versus low switch probability items) which acts as a cue to trigger proper control states (Braver, 2012; Chiu and Egner, 2017), we hypothesized that the *transient*, item-specific valence could be salient and effective in modulating reactive metacontrol (i.e., the ISSP effect). With that regard, we tested if the ISSP effect is modulated by the transient, item-specific valence, to address whether metacontrol is triggered by aversiveness or simply control demand variations. If the ISSP effect is significant without being modulated by the transient negative valence, it supports the mere-experience hypothesis such that the control demand changes are enough for metacontrol and aversiveness does not benefit metacontrol. However, the affective-signaling hypothesis is supported, if the ISSP effect is modulated by the transient background valence. This experiment was pre-registered at https://aspredicted.org/9C5_PDC. Beyond addressing the primary question, given Experiment 1’s exploratory findings, we were mindful of the potential gender difference in the interaction between background valence and reactive metacontrol. We, therefore, sufficiently powered our study to allow for subsequent analyses using a gender variable.

### Method

#### Participants

Seven hundred and nineteen Purdue University undergraduate students (*M_age_* = 18.9, *SD_age_* = 1.3, 358 females, 361 males) submitted informed consent to participate in this experiment for 2 credits in return. The participant’s gender was determined by a forced-choice binary question (male or female). This study was approved by the Purdue University Institutional Review Board. To determine sample size, we built a multivariate normal distribution with the ISSP effect interaction scores obtained in Experiment 1 female dataset (i.e., negative-valence group: M_ISSP_ = 29.66, SD_ISSP_ = 57.76; positive-valence group: M_ISSP_ = 10.48, SD_ISSP_ = 60.64). Setting the type I error = 0.05 and power = 0.85, the smallest sample size for each valence group was 166 to detect the effect of valence modulating the ISSP interaction score. The simulation procedure was implemented in R by randomly drawing samples from a multivariate normal distribution built by the ISSP effect in the negative-valence group and the ISSP effect in the positive-valence group obtained in Experiment 1. The final analysis included data from the 682 participants, female (negative: 168, positive: 170), male (negative: 174, positive: 170), after excluding outlier participants who were outside of the range of ± 2 SD from the group’s mean accuracy. The 1st and 3rd quartile accuracy scores from the sample size 682 were 82% and 90%. Note that our main focus was on examining metacontrol in two background valences with an entire sample, however, we collected enough sample size to examine it in two genders separately.

#### Stimuli

We used a subset of 32 full-color positive- (*M*_valence_ = 7.3, *M*_arousal_ = 4.6) and negative- (*M*_valence_ = 3.6, *M*_arousal_ = 6.0) valence NAPS images, which were rated by the database contributors. The set contained an equal number of images (8 images, 4 positive images and 4 negative images) in the four categories (i.e., a female adult, a male adult, a female child, or a male child). For each participant, 8 images (2 from each of the four categories) were randomly chosen and used. In this experiment, we included both response-incompatible and response-compatible categories because we only had one metacontrol condition (reactive only). The images were displayed with a size of 499 pixels in width and 500 pixels in height. A different set of 8 images were used exclusively in the practice session.

#### Design and procedure

As our interest here is reactive metacontrol, Experiment 2 consists of one unbiased switch probability list of 560 trials with an item-specific switch probability (ISSP) design. Half of the items were the low switch probability items associated with a 10% chance of switching (4 items; 252 repeat trials; 28 switch trials), and the other half were the high switch probability items associated with a 90% chance of switching (4 items; 28 repeat trials; 252 switch trials). A total of 8 unique items were provided. In Experiment 1, the inducer items in the low and high switch probability lists (i.e., 120 trials each in two lists, a total of 240 trials) were reappeared in the last medium, unbiased switch probability list to facilitate item-specific learning (i.e., reactive metacontrol). To ensure a similar amount of learning as in Experiment 1, the first 240 trials were treated as burn-in trials and not included in the main analyses. The main, practice trial progression and categorization tasks were the same as in Experiment 1. Experiment 2 was also conducted online. Different from Experiment 1, at the end of Experiment 2, we asked the participants to rate the entire stimulus set (32 images) on a Likert scale from 1 (very negative) to 7 (very positive) to ensure that the negative images are indeed perceived as negative in our sample. The images were rated after the task-switching trials.

Notably, we manipulated the *valence* (positive, negative) *between-stimuli* (i.e., high versus low switch probability items), while holding the overall background valence neutral within each participant. There were two different pairings of switch probability with valence, and each participant was assigned to only one pairing. Approximately half of the participants were assigned to the pairing of “high+/low-” and performed the task with high switch probability items that are positively valenced (+), and low switch probability items that are negatively valenced (−; Figure 1B, top row). By contrast, the others were assigned to the pairing of “high−/low+” and performed the task with high switch probability items that are negatively valenced (−), and low switch probability items that are positively valenced (+; Figure 1B, bottom row). In sum, on a trial-by-trial basis, participants encountered either a high or a low switch probability item, demanding reactive metacontrol, while at the same time processing either a positive or a negative image, creating a transient, background valence.

#### Data analysis

We examined if the ISSP effect is modulated by different pairings of valence and item switch probability. As in Experiment 1, to index reactive metacontrol, we first calculated low, high switch probability items’ switch costs and then the difference between two items’ SC. With the entire sample, we performed a one-sample t-test (against μ = 0) on the reactive metacontrol score to test whether the ISSP effect was significant. We then compared the reactive metacontrol scores between the high+/low-group and the high−/low+ group using a two-tailed two independent samples Welch’s unequal variances *t*-test. As in Experiment 1, we used the Welch *t*-test statistics which do not require equal variances between the two groups, and reported the two-tailed results. We followed the same trial exclusion procedure and reporting material (e.g., M, SD, *t*, Cohen’s *d*, BF*_01_*) as in Experiment 1.

### Results

#### Valence ratings

The negative images (M = 2.4, SD = 0.6) were indeed rated to be more negative than the positive images (M = 6.0, SD = 0.6), *t*(681) = −95.44, *p* < 0.001. As we used a 7-point scale as opposed to a 9-point scale in the original NAPS study, we did a linear transformation of the means in order to compare our results with the original means. After transformation, the mean ratings were 3.1 for the negative images and 7.7 for the positive ones, which were comparable to the means provided by the NAPS database (negative: 3.6; positive: 7.3).

#### Reactive Metacontrol

##### Response times

**Figure 5:**
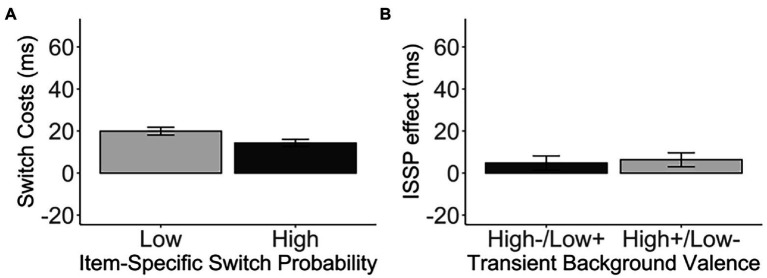
**(A)** The switch costs (in ms) of the low and high switch probability items in the medium, unbiased list-wide switch probability list. **(B)** The ISSP effect (in ms) as a function of the transient background valence. Error bars indicate the standard error of the mean.

As expected, we observed a significant ISSP effect, *t*(681) = 2.35, *p* = 0.019, *d* = 0.09, 95% CI [0.92, 10.26], BF_01_ = 1.50 (Figure 5A). That is, switch costs were reduced in the high switch probability items (M = 14.35, SD = 43.38) compared to the low switch probability items (M = 19.95, SD = 49.15) intermixed in the same, switch-probability unbiased list. However, the *valence* did not modulate the ISSP effect, *t*(679.76) = 2.35, *p* = 0.746, *d* = 0.02, 95% CI [−7.80, 10.88], BF_01_ = 11.12 (high−/low+: M = 4.82, SD = 62.89, high+/low-: M = 6.36, SD = 61.35; Figure 5B, Table 5).

**Table 5:** Mean response times (ms) with standard deviations in Experiment 2 as a function of background valence, switch probability, and transition.

Switch probability	Transition	High−/Low+	High+/Low−
		*N* = 342	*N* = 340
Low items	Repeat	643 (82)	653 (77)
	Switch	662 (99)	674 (95)
High items	Repeat	643 (90)	654 (88)
	Switch	657 (88)	668 (84)

Low and high items refer to the low and high switch probability items in the medium (50%) list-wide switch probability list. *N* represents the sample size in each group.

##### Accuracy

The ISSP effect was not significant, *t*(681) = 0.99, *p* = 0.323, *d* = 0.04, 95% CI [−0.46, 1.40], BF_01_ = 14.26. The ISSP effect was neither modulated by *valence*, *t*(678.39) = 1.23, *p* = 0.220, *d* = 0.09, 95% CI [−0.70, 3.03], BF_01_ = 5.59 (high−/low+: M = −0.11, SD = 12.14, high+/low-: M = 1.06, SD = 12.67). See Table 6.

**Table 6:** Mean accuracy (%) with standard deviations in Experiment 2 as a function of background valence, switch probability, and transition.

Switch probability	Transition	High−/Low+	High+/Low−
		*N* = 342	*N* = 340
Low items	Repeat	87 (10)	89 (8)
	Switch	84 (14)	87 (11)
High items	Repeat	88 (12)	88 (12)
	Switch	85 (11)	87 (9)

Low and high items refer to the low and high switch probability items in the medium (50%) list-wide switch probability list. *N* represents the sample size in each group.

#### Gender difference analysis

We furthermore examined a possible gender difference based on Experiment 1’s exploratory analysis result where we observed a pattern of the ISSP effect being modulated by background valence only in the female sample. Note that Experiment 2 has enough power to detect a valence effect on the ISSP effect in each gender.

As in the previous analysis, we computed the reactive metacontrol scores, i.e., switch cost difference in the high vs. low switch probability items, and compared the scores between the high−/low+ and high+/low− groups. Importantly, the comparison was computed separately for the female and the male sample. We computed a two-tailed two independent samples Welch’s unequal variances *t*-test and applied the Bonferroni corrections to 2 multiple comparisons. We followed the same trial exclusion procedure and reporting material (e.g., M, SD, *t*, Cohen’s *d*, BF*_01_*) as in Experiment 1.

We also reported results from analyzing data with a 2 (item-specific switch probability: low, high) × 2 (transition: repeat, switch) × 2 (background valence: positive, negative) x 2 (gender: female, male) mixed-design rmANOVA at http://doi.org/10.17605/OSF.IO/2BDVC.

#### Reactive metacontrol

##### Response times

**Figure 6:**
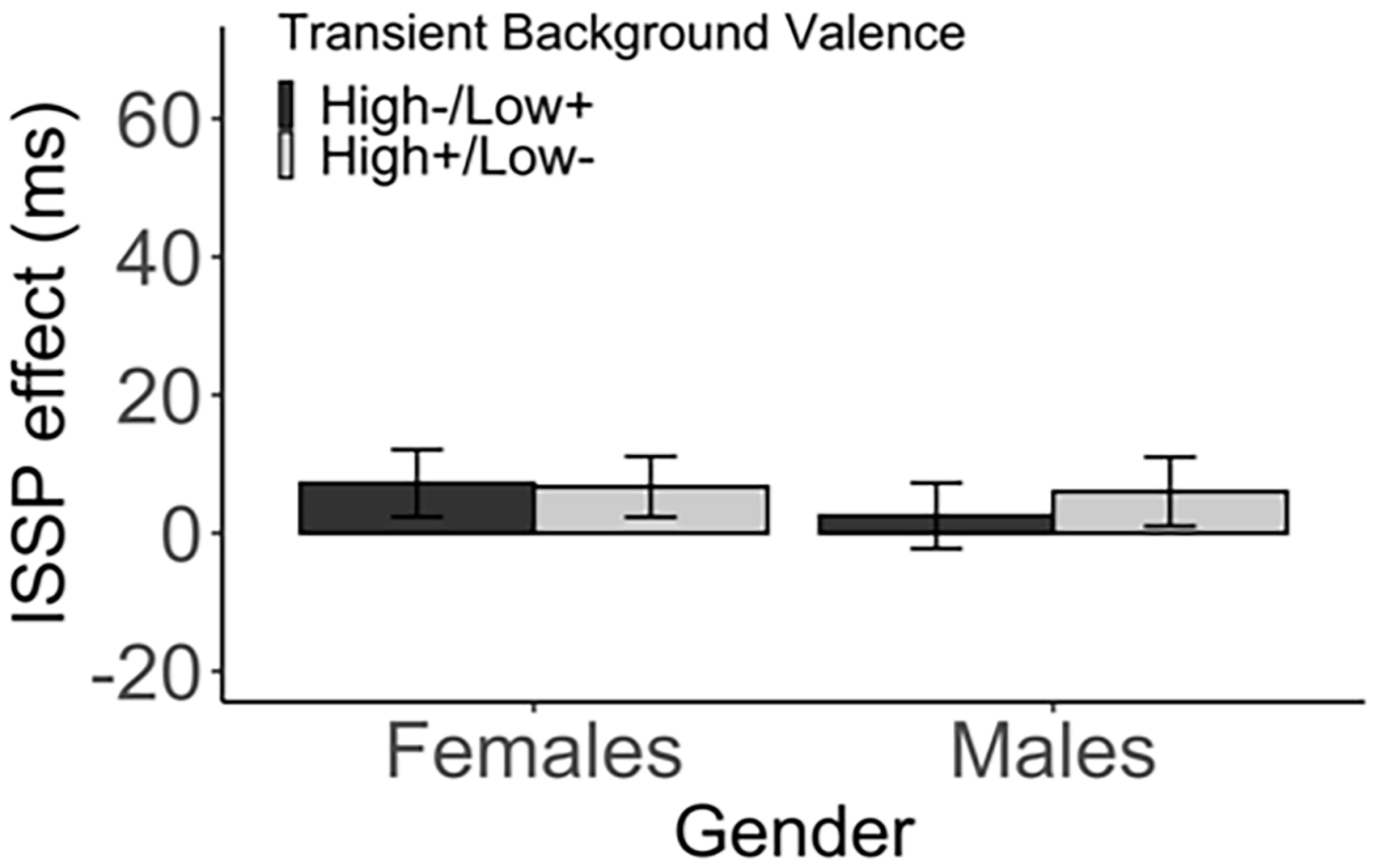
The ISSP effect (in ms) as a function of the transient background valence and gender. Error bars indicate the standard error of the mean.

The *valence* did not modulate the ISSP effect in both genders. In female participants, there was no difference in the ISSP effect between the two groups that received different pairings of switch probability with valence (high−/low+ vs. high+/low−), *t*(332.19) = 0.08, *p* > 1 (corrected), *d* < 0.01, 95% CI [−12.40, 13.42], BF_01_ = 8.91 (high−/low+: M = 7.22, SD = 63.12, high+/low−: M = 6.71, SD = 57.36). Likewise, there was no difference between the two male groups, *t*(340.68) = −0.51, *p* > 1 (corrected), *d* = 0.05, 95% CI [−17.09, 10.08], BF_01_ = 7.42 (high−/low+: M = 2.51, SD = 62.76; high+/low−: M = 6.01, SD = 62.26; Figure 6; Table 7).

**Table 7:** Mean response times (ms) with standard deviations in Experiment 2 as a function of background valence, switch probability, and transition separately in males and females.

		High−/Low+		High+/Low−	
Switch probability	Transition	Females	Males	Females	Males
		*N* = 168	*N* = 174	*N* = 170	*N* = 170
Low items	repeat	636 (80)	650 (83)	654 (61)	652 (90)
	switch	655 (101)	669 (97)	675 (79)	673 (109)
High items	repeat	639 (91)	646 (89)	657 (74)	651 (100)
	switch	651 (88)	663 (88)	671 (66)	665 (98)

Low and high items refer to the low and high switch probability items in the medium (50%) list-wide switch probability list. *N* represents the sample size in each group.

##### Accuracy

The ISSP effect was not modulated by *valence* in female participants, *t*(335.96) = −1.06, *p* = 0.578 (corrected), *d* = 0.12, 95% CI [−4.29, 1.28], BF_01_ = 4.85 (high−/low+: M = −0.14, SD = 13.00, high+/low−: M = 1.36, SD = 13.02), nor in male participants, *t*(337.67) = −0.62, *p* > 1 (corrected), *d* = 0.07, 95% CI [−3.34, 1.68], BF_01_ = 6.85 (high−/low+: M = −0.08, SD = 11.27, high+/low−: M = 0.75, SD = 12.34). See Table 8.

**Table 8:** Mean accuracy (%) with standard deviations in Experiment 2 as a function of background valence, switch probability, and transition separately in males and females.

		High−/Low+		High+/Low−	
Switch probability	Transition	Females	Males	Females	Males
		*N* = 168	*N* = 174	*N* = 170	*N* = 170
Low items	Repeat	86 (10)	87 (10)	89 (8)	88 (9)
	Switch	84 (14)	85 (13)	87 (11)	86 (12)
High items	Repeat	87 (11)	88 (13)	88 (12)	88 (11)
	Switch	85 (10)	85 (11)	87 (8)	86 (9)

Low and high items refer to the low and high switch probability items in the medium (50%) list-wide switch probability list. *N* represents the sample size in each group.

### Discussion

In Experiment 2, while we observed a significant ISSP effect, the ISSP effect was not modulated by the transient background valence. Namely, the ISSP effect was not modulated when negative valence was added to the high switch probability items, which could induce negative valence themselves because of the frequent task switches. We did not observe any gender difference in the ISSP modulation effect by valence. In sum, findings in Experiment 2 suggest that variation in control demands is sufficient to drive reactive metacontrol, and aversiveness does not benefit reactive metacontrol.

## General discussion

While recent studies have documented different types of metacontrol mediated by various cues (e.g., item, list) in a wide variety of paradigms (e.g., a Stroop, a task-switching, a flanker paradigm; Gonthier et al., 2016; Bugg and Gonthier, 2020; Kang and Chiu, 2021), it remains unclear what drives metacontrol behaviors in the first place. Here, we address this question indirectly by examining whether the valence of the background in which metacontrol occurs modulates metacontrol. In Experiment 1, we used a task-switching paradigm previously established in Kang and Chiu (2021) to induce proactive and reactive metacontrol and indexed them with the LWSP and ISSP effect, respectively. Moreover, we induced the sustained background valence to examine if it would modulate the LWSP and the ISSP effects. In Experiment 2, we only induced reactive metacontrol, the ISSP effect, using the same task-switching paradigm. We added the transient background valence on to reactive metacontrol to examine if it would modulate the ISSP effect. Overall, while we were able to replicate the LWSP effect in Experiment 1 and the ISSP effect in Experiment 2, we did not find them to be modulated by valence, when the background valence being a sustained (Experiment 1) or a transient one (Experiment 2). That is, the negative background did not enhance the LWSP/ISSP effects.

According to the affective-signaling hypothesis (Dignath et al., 2020), conflicts incur aversiveness and aversiveness *per se* drives metacontrol. While conflicts in different cognitive paradigms have been reported to incur negative affect (Dreisbach and Fischer, 2012; Braem et al., 2017; Vermeylen et al., 2019), the studies examining how metacontrol is modulated by background valence has not resulted in consistent findings. Some studies reported enhanced metacontrol in a negative background (van Steenbergen et al., 2009, 2010) whereas, others reported enhanced metacontrol in a positive background (Dreisbach et al., 2018, 2019). Among the former ones, for example, van Steenbergen et al. (2009) inserted sad faces or smiley faces after each trial in a flanker task. These faces could be perceived as feedback on each trial, however, participants were informed that the faces are not performance contingent. Instead, the faces were simply intended to induce a transient negative or positive affect and are orthogonal to the congruency sequence effect (CSE). van Steenbergen et al. (2009) found that metacontrol (i.e., CSE in a flanker task) was enhanced in the negative valence condition where sad faces were presented in-between trials. By contrast, it was reduced in a positive valence condition where smiley faces were presented in-between trials, which is likely due to the positive affect counteracting the aversiveness of recently-encountered conflicts. Similarly, using a mood induction procedure to manipulate the background valence, van Steenbergen et al. (2010) found that metacontrol was enhanced in participants who were in an anxious/sad mood than in those in a happy/calm mood.

In contrast, different findings (i.e., enhanced metacontrol in a positive background) have been documented in some studies, however, by taking a very different approach. More specifically, Dreisbach et al. (2019) manipulated positive and negative valence backgrounds “between-items” that were associated with mostly congruent versus mostly incongruent trials, in a Simon task. Specifically, one group of subjects saw positive images pairing with mostly congruent items, and negative images pairing with mostly incongruent items. Whereas, the other group saw the opposite valence-item pairings. Note that their Simon task required participants to categorize an image presented either to the left or to the right of the center according to a rule (e.g., animal vs. human). Therefore, the valence of an image was completely task-irrelevant. Dreisbach et al. (2019) found enhanced metacontrol in the mostly incongruent items as compared to the mostly congruent items, but this effect was more evident in the group where the mostly incongruent items were positively valenced. Thus, this finding supports the idea that the aversiveness of conflicts looms larger when the conflicts are detected or processed in a positive background. In sum, the findings are mixed regarding how affective information processing interacts with metacontrol.

The current study took metacontrol modes and temporal length of background valence into consideration and supported that negative affect or aversiveness may not modulate metacontrol. This is consistent with the computational modeling studies on metacontrol (Botvinick et al., 2001; Blais et al., 2007; Jiang et al., 2014). Specifically, Botvinick et al. (2001) suggested a mechanism of metacontrol such that conflicts would bias the task-relevant pathway over the task-irrelevant one. For example, it was demonstrated that incongruent trials in a Stroop paradigm (e.g., a word RED in blue color in a task of reading a color) would bias a color processing pathway. It implies that contexts associated with frequent conflicts would produce better performance (e.g., reduced switch costs) by the accentuated task-relevant pathway and the attenuated task-irrelevant one within the context. Blais et al. (2007) extended the model from a pathway level to an item level and explained metacontrol. Specifically, the model explained that contexts with frequent conflicts would result in better performance by the biased task-relevant specific items. For example, when a word RED is often presented in a blue color, a RED in blue would bias a color blue instead of biasing a color processing pathway. Together, these modeling studies support the idea that when control demands are enhanced, a relevant system (e.g., anterior cingulate cortex, ACC) associates the context with the task-relevant components (either pathways or specific items) to better resolve the conflicts within the context. These studies successfully simulated metacontrol. Our results, therefore, add to this body of literature and support the mere-experience hypothesis as we showed that metacontrol is sufficiently triggered by the presence of control demand changes and that adding aversiveness to the background of control processing does not modulate metacontrol.

However, we found a significant ISSP effect in female participants that were assigned to the sustained, negative background, but neither in female participants that were assigned to the sustained, positive background nor in any male participants altogether. This finding is only partially predicted by the affective-signaling hypothesis in that if an individual is responsive to aversiveness, aversiveness could drive metacontrol. The observation is consistent with the previous studies as in van Steenbergen et al. (2009, 2010) which showed negative affect benefitting metacontrol. Extrapolating from the single case where we observed an enhanced metacontrol, we suspect that there are at least two prerequisites for it to happen. The first is that metacontrol occurs in a sustained, negative (aversive) background. This is based on the finding that we only found valence modulating metacontrol in Experiment 1 but not in Experiment 2 where the overall background was neutral within each participant. When the positive images were presented along with negative images as in our Experiment 2, the aversiveness of the negative images can be weakened and neutralized. Whereas, when the negative images are presented throughout an experimental session, the negative valence could add up, intensifying aversiveness, which in turn benefits metacontrol. The second is that metacontrol relies on reactive processing. In our case, the ISSP effect relies critically on “reactive” processing, which is likely achieved by increased attention to stimulus-level information on each trial. This increased attention could help or enhance the processing of valence because valance is in fact task-irrelevant in our experiment (i.e., participants categorize stimuli according to gender and age of the main character in the images). With increased attention to each stimulus’s negative valence, the ISSP effect might be enhanced due to the intensified aversiveness. On the other hand, the LWSP effect relies on proactive processing, which might decrease attention to task-irrelevant valence information and reduce any effect the valence might confer. As a result, proactive metacontrol might not be enhanced by adding any task-irrelevant aversiveness. A similar observation has been documented (Cañadas et al., 2016; Dreisbach et al., 2019; Zhang et al., 2019) as they also did not find valence modulating metacontrol when it relies on proactive processing. In sum, we suspect that reactive metacontrol (but not proactive metacontrol) may be enhanced in a sustained aversive background. Future studies are required to replicate this finding in different paradigms.

Our exploratory finding of valence modulating the ISSP effect only in female participants suggests that there might be a gender difference in the extent to which valence interacts with control processing. This could be related to similar gender differences in affective processing that have been documented in the literature. For example, female participants have been found to be more responsive to stimuli with a negative valence (Wrase et al., 2003; Kemp et al., 2004; Noon et al., 2019; Costa et al., 2022). In Kemp et al. (2004), participants were required to rate how unpleasant/pleasant an image was after viewing one of the negative, positive, or neutral images (from the International Affective Picture System, IAPS) on each trial. They adopted a steady-state probe topography (SSPT) technique where a rapid and repetitive visual flicker is presented during electroencephalogram (EEG) recording. The latency between the valence image induced responses and the flicker induced oscillatory responses (i.e., steady-state visually evoked potential, SSVEP) was the main dependent variable. Interestingly, Kemp and colleagues observed a latency reduction in the frontal region on the negative images (compared with the positive-valence one) only in female participants but not in male participants. This frontal latency reduction has been linked to regulatory processes in response to the negative affect. Thus, their finding of a gender difference suggests that negative images might have a greater impact on female than on male participants, requiring them to initiate a regulatory process to counteract the induced negative affect. Shackman et al. (2011) have proposed that the anterior midcingulate cortex (aMCC) might be responsible for regulating both cognitive control and affect. According to this proposal, aversiveness could enhance metacontrol simply by co-activating the common ACC hub. Putting these findings together, one explanation of our finding is that female participants were more likely to exhibit the enhanced ISSP effect because their ACC was co-activated by both the affect regulation and control demand regulations. However, we failed to show a statistically significant difference in reactive metacontrol from sustained negative vs. positive background, and the idea that activations in ACC play a key role in mediating females’ enhanced reactive metacontrol remains to be tested in a formal neuroimaging study.

One thing to note is that the ISSP effect with the entire sample failed to reach significance in Experiment 1 (*p* = 0.061). As for the Experiment 1, we suspect that the lack of significance was due to the fixed order with the ISSP manipulation embedded in the last list. This might have contributed to the ceiling effect of performance in the last list supported by relatively faster RT in the last list inducing the ISSP effect (M = 708) compared with the RT in the first two lists inducing the LWSP effect (M = 719). However, we were able to replicate the ISSP effect with faster RT in Experiment 2 than in Experiment 1 (681 vs. 713), which complicated the ceiling effect explanation. Nonetheless, we caution against directly comparing RT across these two experiments, given many differences between the two (i.e., participants in Experiment 2 did not do the high/low lists and they are in an overall ‘neural’ background valence). The observed ISSP effect in Experiment 1 from the entire sample showed a trend toward significance and the ISSP effect of the female participants in the sustained negative background was significant after multiple-comparison corrections. Additionally, the LWSP effect with inducer items showed a numerically larger LWSP effect (M = 45.99 ms) than the LWSP effect with diagnostic items (M = 41.27 ms), without a significant modulation effect of valence, *t*(269.73) = 1.43, *p* = 0.15. Since the same inducer items were used across the LWSP and ISSP effects, we suspect that the inducer items indeed produced the reactive metacontrol effect, while small in size. Therefore, we are cautious to conclude the overall ISSP pattern in Experiment 1 as a failure of replication but as an intriguing case that motivates future studies. Future studies might shed light on whether there is a reliable gender difference by focusing on the reactive metacontrol in a sustained negative background with a different age/demographic makeup (given that ours is a homogenous college undergraduate population).

Given our design, one might be concerned that the affective valence of images can become habituated over repeated exposure. However, in Experiment 2, participants rated the images after repeated exposure to them in the switching task and we still observed a significant difference in valence ratings of positive vs. negative images. Furthermore, reactive metacontrol was assessed during the second half of the experiment but the impact of valence on it was nonetheless detected in females. Therefore, we think that the valence images in Experiment 1 likely maintained their affective value throughout the task. While the affective images were perceived as indeed negative or positive, we cannot exclude the possibility that the affective picture system (i.e., NAPS) does not interact optimally with our cognitive task (i.e., cued-task switching paradigm). Given the use of a small set of stimuli and only response incompatible items in Experiment 1, one might be concerned that the reported LWSP/ISSP effects are not driven by higher-level metacontrol but by lower-level stimulus–response learning. We note that we adopted an identical design (except for adding the background valence implementation) as in Kang and Chiu (2021) where we showed that the LWSP and ISSP effects were not dependent on the lower-level stimulus–response learning. Specifically, we reported similar LWSP and ISSP effects between response compatible (i.e., items requiring the same response in two tasks, therefore, susceptible to the lower-level stimulus–response learning) and response incompatible items (Kang and Chiu, 2021). Relatedly, Whitehead et al. (2017) used a small set of 4 items repeatedly and observed an item-specific proportion congruency effect (indexing reactive metacontrol) in terms of an EEG signature (i.e., frontocentral N2, negative deflection around 200–300 ms after the stimulus presentation observed in the frontocentral electrodes). Since the frontocentral N2 is thought to reflect a control-related component originated from the ACC (as opposed to a visual attention-related component observed in posterior N2; Van Veen and Carter, 2002; Folstein and Van Petten, 2008), the study suggests that the limited number of items do not necessarily produce lower-level, stimulus–response learning.

Our study has a limitation where we only used images from the NAPS database. Especially, NAPS valence images induced greater arousal as the images are more negatively valenced, Pearson’s *r* = −0.80, *t*(370) = −26.03, *p* < 0.001, as supported by the significant correlation between arousal and valence of face valence images in NAPS. Accordingly, arousal was unmatched between the negative and positive valence conditions in our study. Assuming that negative images with higher arousal should be more aversive than those with lower arousal, our stimuli should favor the finding of valence modulating metacontrol. Yet, we failed to find such an effect. However, future studies should more systematically study the impact of arousal separated from valence on metacontrol. Our study did not distinguish stimuli-driven aversiveness from conflict-triggered aversiveness. It is based on the reason that aversiveness, regardless of its source, would be managed by shared systems. However, one would need to objectively measure or index conflict-triggered aversiveness and see how that might modulate metacontrol. Lastly, although we have incorporated neuroimaging findings to interpret our findings, we do not have direct evidence. There may be other sources of individual propensities worth considering that might contribute to the differences in sensitivity to valence manipulation. That is, our study posited a possible gender difference in the interaction between affective and metacontrol processing, however, we acknowledge that these inferences have limitations.

## Conclusion

The associative learning guided metacontrol has been getting attention due to its’ optimal functioning of providing flexible but also fast, energy-efficient control instantiations. These benefits lead to the question of what drives metacontrol in the first place. Our two experiments examined two related hypotheses: the mere-experience vs. the affective-signaling hypothesis of metacontrol. Our data supports the mere-experience hypothesis that control demand changes are sufficient to incur metacontrol.

## Data availability statement

The datasets presented in this study can be found in online repositories at: http://doi.org/10.17605/OSF.IO/2BDVC.

## Ethics statement

The studies involving human participants were reviewed and approved by Purdue University Institutional Review Board. The patients/participants provided their written informed consent to participate in this study.

## Author contributions

MK and CY-C conceived the study design. MK programmed the experiments, collected and analyzed the data, and wrote the initial draft of the manuscript. All authors contributed to the article and approved the submitted version.

## Conflict of interest

The authors declare that the research was conducted in the absence of any commercial or financial relationships that could be construed as a potential conflict of interest.

## Publisher’s note

All claims expressed in this article are solely those of the authors and do not necessarily represent those of their affiliated organizations, or those of the publisher, the editors and the reviewers. Any product that may be evaluated in this article, or claim that may be made by its manufacturer, is not guaranteed or endorsed by the publisher.

## Footnotes

^1^ https://exp.lobi.nencki.gov.pl/dnaps
